# Association between Food-Specific Immunoglobulin G_4_ Antibodies in Adults with Self-Reported Signs and Symptoms Attributed to Adverse Reactions to Foodstuffs

**DOI:** 10.3390/biomedicines11123335

**Published:** 2023-12-17

**Authors:** Lisset Pantoja-Arévalo, Eva Gesteiro, Torsten Matthias, Rafael Urrialde, Marcela González-Gross

**Affiliations:** 1 ImFINE Research Group, Department of Health and Human Performance, Universidad Politécnica de Madrid, 28040 Madrid, Spain; eva.gesteiro@upm.es (E.G.); marcela.gonzalez.gross@upm.es (M.G.-G.); 2 EXERNET Spanish Research Network on Physical Exercise and Health, Universidad de Zaragoza, 50009 Zaragoza, Spain; 3 Department of Research and Development, Aesku.Diagnostics GmbH, 55234 Wendelsheim, Germany; 4 Department of Genetics, Physiology and Microbiology, Universidad Complutense de Madrid, 28040 Madrid, Spain; 5 Department of Pharmaceutical and Health Sciences, Universidad San Pablo CEU, 28040 Madrid, Spain; 6 Biomedical Research Centre of Pathophysiology, Obesity and Nutrition-CIBERobn, Carlos III Health Institute, 28040 Madrid, Spain

**Keywords:** adverse effects, allergens, antibodies, food hypersensitivity, immunoglobulin G, point-of-care testing, signs and symptoms

## Abstract

Signs and symptoms attributed to adverse reactions to foodstuffs (ARFS) need tools for research and evaluation in clinical practice. The objectives of this study were (a) to evaluate the most frequent self-reported signs and symptoms attributed to ARFS in Spanish adults, (b) to determine the prevalence of food-specific IgG_4_ antibody reactions (AbRs), and (c) to investigate the association between self-reported ARFS symptomatology and food-specific IgG_4_ AbRs. Food-specific IgG_4_ AbRs against 57 common food and beverages (AESKUCARE-T2FA^®^ in vitro point-of-care test kit, Aesku.Diagnostics GmbH, Germany) were determined in capillary blood samples of 205 volunteers living in the Region of Madrid (Spain). The most frequent self-reported signs and symptoms were related to skin (43%), digestive (41%), and nervous system (NS, 33%) problems. The prevalence of food-specific IgG_4_ AbRs was cow’s milk (73%), sheep’s milk (70%), casein (66%), and goat’s milk (56.10%). Positive IgG_4_ AbRs against tomato had a profile consisting of 3/4 of skin problems, more than half of digestive, and 2/5 of NS self-reported signs and symptoms. In conclusion, at least 1/3 of the studied sample reported skin, digestive, and NS signs and symptoms. The most frequent food-specific IgG_4_ AbRs were related to dairy. Skin problems were more frequent in positive tomato IgG_4_ AbRs.

## 1. Introduction

Immune reactions to food-specific allergens can be indicative of adverse reactions to foodstuffs (ARFS), a significant cause of morbidity worldwide and a considerable burden on current public health in both developed countries and emerging economies [1,2,3]. ARFS refers to any abnormal reaction to the ingestion of food products and their byproducts (e.g., casein, lactose). These food reactions include, among others, food allergy (FA) and food intolerance (FI) reactions [4]. The scarcity of precise data makes it difficult to estimate the prevalence of FA and FI among the world population. Some studies have indicated 2%, raising to more than 30%, respectively [5,6,7]. Different diagnostic tools have been used over the years: immunology-based tests and other clinical or blood tests, skin prick tests (SPTs), self-reported questionnaires, and double-blind placebo-controlled food challenges (DBPCFCs), which are currently considered the gold standard method to determine FA and FI [8]. 

Food antigens can enhance gastric acid secretion and alter the gastrointestinal (GI) mucosa, which subsequently increases its permeability to food antigens. The production of large quantities (>3.4 kU_A_/L) of positive food-specific immunoglobulin E (IgE) antibodies may not be sufficient for a good model of GI allergy, as certain IgG subclasses (IgG_4_ in humans) can also bind to and activate mast cells [9]. Clinical observations have suggested that food-specific IgG antibody subclasses involved in type III reactions may initiate some ARFS, permeability, and chronic intestinal inflammations [10]. Meanwhile, some other studies suggest that IgG_4_ could be related to improving the prediction of DBPCFC outcomes [5].

Signs and symptoms of FA can start within minutes of exposure (commonly type I or IgE-mediated responses) to a trigger food and any combination of local oral, dermatological, GI, or respiratory signs and symptoms can occur [6]. Meanwhile, the symptomatology of some FI (IgG, IgG_4,_ or other classes and non-immune-mediated responses) cannot be easily explained by the currently understood biological processes, except for fructose and lactose intolerance, which have been correlated with signs and symptoms of different functional GI disorders (FGIDs) [6,11]. IgG_4_-mediated responses have been associated with a wide range of specific and non-specific symptomatology [12] and implicated in signs and symptoms related to allergies such as rashes, urticaria, and asthma, but also signs and symptoms of the gastrointestinal tract (mostly suggestive of irritable bowel syndrome (IBS), including abdominal cramps, diarrhea, and constipation [12]. Manifestations such as migraines, chronic fatigue, and hair loss have also been reported [12,13]. Therefore, a detailed study of the most frequent self-reported signs and symptoms attributed to ARFS and the association with IgG_4_ antibody reactions (AbRs) may contribute and provide a wider vision of the role of IgG_4_. 

Today, it is recommended to analyze an open panel of food and beverages’ allergens and allergen mix in the Mediterranean zone. A continuous change in dietary patterns toward a more Western diet has been observed in Mediterranean areas. Due to the current globalization of crops, there are Mediterranean areas in which typical foods from other regions are grown, and which are also part of the current frequent consumption of the Mediterranean diet (MD). As a consequence, the influence of other cultures (Asian, e.g., soja, kiwi; African, e.g., pineapple; and American, e.g., chocolate) has been responsible for some of the recent changes in the food intake in the Spanish population when choosing food [14,15]. 

The aims of this study were (a) to evaluate the most frequent self-reported signs and symptoms attributed to ARFS in Spanish adults, (b) to determine the prevalence of food-specific IgG_4_ antibody reactions (AbRs), and (c) to investigate the association between self-reported ARFS symptomatology and food-specific IgG_4_ AbRs. 

## 2. Materials and Methods

This is a cross-sectional descriptive study conducted from October 2017 to October 2019 in point-of-care (POC) health centers, such as medical health centers and pharmacies in the city of Madrid and surrounding villages of the Region of Madrid (Spain). Informed consent was obtained from all subjects involved in the study. All subjects were informed about the goals of the study and agreed that their clinical data could be used for research purposes [16,17]. Data were managed using a participant code to preserve the anonymity of all subjects. This study was carried out according to the principles of the Declaration of Helsinki and following the Spanish and European regulations regarding data protection [18]. The protocol has been approved by the Ethics Committee of the Universidad Politécnica de Madrid (reference number 20200602) and registered on ClinicalTrials.gov (Clinical Trials ID NCT05681975 and protocol ID 1720IL0389). 

Subjects were enrolled in their nearest POC health center during working hours from 9 a.m. to 9 p.m. The inclusion criteria were adults (>18 years old) who presented self-reported signs and symptoms related to ARFS. The exclusion criteria were adults with previous medical diagnoses of a positive FA and/or FI, adults receiving antibiotic treatment, and those living outside of the region of Madrid, Spain. 

Subjects registered their self-reported signs and symptoms through a generally adapted anamnesis form, which included a list of the main signs and symptoms of a FA recommended in the ‘Guidelines for the use and interpretation of diagnostic methods in adult food allergy’ [19]. A blank space was also provided for participants to register any additional signs or symptoms not included in the provided list. Self-reported signs and symptoms were classified into the 3 most reported categories: skin and subcutaneous tissue (or dermatological, CIE-10: L00-L99), digestive (or GI, CIE-10: K00-K93), and nervous system (NS, CIE-10: G00-G99) problems using the ICD-10 or CIE-10 classification to establish the human body systems involved in the studied sample [20]. 

Food-specific IgG_4_ AbRs against 57 common food antigens of the current Spanish eating patterns were analyzed in 50 μL of capillary blood obtained from the fingertip using a 7157 safety lancet (HTL-Strefa Inc., Lodz, Poland). Subjects were asked to avoid all 57 foodstuffs from the complete panel for a minimum of 24 to 72 h before the blood sampling. Immunoassay kits, previously NutriSMART^®^ and currently commercialized under AESKUCARE-T2FA^®^ in vitro diagnostic POC test kits (Aesku.Diagnostics GmbH, Wendelsheim, Germany), were used with 3 semiquantitative levels of detection of IgG_4_ Ab (levels 1, 2, and 3 being low, moderate, and high levels, respectively). All levels were compared to the standard control of the manufacturer (IgG_4_). IgG_4_ values ranged from 0.08 to 1259.7 U/mL; values higher than 3.50 U/mL were considered higher than the standard control; 1 U = 1.47 ng. Food-specific IgG_4_ AbRs were considered as Level 1 (low): equal or below the standard control of (IgG_4_); Level 2 (moderate): slightly above the standard control of (IgG_4_); and Level 3 (high): above the standard control of (IgG4). According to the manufacturer, for food-specific IgG_4_ AbR determinations using the AESKUCARE-T2FA^®^ test kit, it is not recommended to consider strictly defined positive population limits, as it is currently established for IgE AbR levels, and because IgG_4_ is a biomarker that is still under research when related to food and beverages’ allergens. Thus, for this study, a positive population response was considered when Levels (2 + 3) were summed up equal to or greater than 50% of the prevalence of the same food, beverage, or food allergen mix (≥50%). Individual positive responses were Level 3 of IgG_4_ AbRs.

The 57 studied food antigens were classified into 40 different wells of the AESKUCARE-T2FA^®^ kit and 40 variables were considered: wheat, rye, barley, oat, grain mix A (buckwheat/amaranth/quinoa mix), grain mix B (corn/rice mix), gluten, peanut, hazelnut, almond, banana, fruit mix A (lemon/orange mix), fruit mix B (strawberry/grape/peach mix), apple, pineapple, kiwi fruit, egg white, egg yolk, casein, cow’s milk, goat’s milk, sheep’s milk, cod, fish mix (salmon/trout mix), tuna, seafood mix (shrimp/squid/octopus mix), tomato, legume mix (peas/green beans mix), vegetable mix A (carrot/celery mix), vegetable mix B (cabbage/broccoli mix), tuber mix (garlic/onion/leek mix), lamb or mutton, meat mix A (pork/beef mix), meat mix B (chicken/turkey mix), potato, soy, yeast mix (baker’s/brewer’s yeast mix), cocoa, coffee, and mustard. 

Body composition was measured using a bioelectrical impedance analysis (BIA) scale, Renpho ES-26M-W (RENPHO, Hong Kong, China), and height was assessed using a standardized stadiometer, InBody BSM170, 35–210 m measure range (InBody Co. Ltd., Seoul, Korea). Self-reported PA was assessed using a modified and adapted version of the International Physical Activity Questionnaire Short Form (IPAQ-SF) and categorized according to the recommendations of the World Health Organization (WHO) [21] and the American College of Sports Medicine (ACSM) guidelines [22,23].

Statistical analysis was performed using SPSS Statistics software (version 25.0, IBM Corp., Armonk, NY, USA). All variables followed the non-normal distribution. A descriptive analysis was performed. Quantitative variables were expressed as mean (M) and standard deviation (SD) and qualitative variables as a percentage (%). Food-specific IgG_4_ AbR levels for 57 different allergens were analyzed as 40 variables, including the allergen mix variables, which were analyzed as one single variable, e.g., legume mix (peas/green beans), fish mix (shrimp, squid, and octopus), etc. The Spearman rank correlation coefficient (rho) was considered to obtain the correlation and association between the food-specific IgG_4_ AbR variables with symptomatology, number of signs and symptoms, body composition, and PA. The non-parametric Kruskal–Wallis test was used to establish if symptomatology was different towards different levels of BMI (underweight, normal weight, overweight, class I obesity, class II obesity, and class III obesity), and also to measure the differences between the 17 most prevalent food-specific IgG_4_ AbRs and the studied age ranges (20–34, 35–49, 50–64, and 65–79-year-old groups). Cut points were established according to quartiles to determine associations with food and beverages’ allergens and their level of IgG_4_ AbRs. The non-parametric Mann–Whitney U test was used for the analysis of the effect of food-specific IgG_4_ AbRs and the number of signs and symptoms over sex. The eta-squared index (η^2^) was used as the effect size and was classified according to Cohen [24] as small significant effect sizes from ≥0.01 to <0.06; medium and significant, from ≥0.06 to <0.14; and large and significant, from ≥0.14. A Chi-squared test was used to analyze whether the clinical profile expressed in the self-reported symptomatology was independent of the level of food-specific IgG_4_ AbRs following the statistical guidelines of the Life Cycle Committee [25]. The level of statistical significance was set at 0.05.

## 3. Results

Two-hundred eleven individuals volunteered for this study; three subjects from outside the Region of Madrid and three subjects under antibiotic treatment were excluded. A total sample of 205 Spanish adults was analyzed: 143 women and 62 men with a mean (±SD) age of 45.5 years old (±14.9 y.o., from 20 to 79 y.o.).

### 3.1. Body Composition

Descriptive data from the sample are shown in Table 1. More than half of the participants were over normal weight (56.59%) and did not meet the WHO recommendations for physical activity (PA) (52.7%).

### 3.2. Signs and Symptoms

The most frequent self-reported signs and symptoms attributed to ARFS in the studied sample were skin and subcutaneous tissue (43.27%), digestive (40.74%), and those related to NS (33.33%) problems. When comparing these signs and symptoms by sex (Table 1), in women, the most common were skin and subcutaneous tissue problems (eczema, psoriasis, dermatitis, and/or acne), followed by digestive self-reported signs and symptoms (abdominal bloating, heartburn, nausea, and/or gastritis), followed by NS problems (body pain and heaviness (back, legs, arms, lower back, facial, jaw and/or joint)). In contrast, the most frequent self-reported signs and symptoms in men were NS problems (depression, anxiety, fatigue, and/or lack of energy), followed by digestive self-reported signs and symptoms (abdominal bloating, heartburn, nausea, and/or gastritis), and lastly, skin and subcutaneous tissue problems (eczema, psoriasis, dermatitis and/or acne). 

Significant differences were observed in the frequency of the reported subcategories of signs and symptoms according to BMI values. There was a significantly higher frequency of subjects who reported a mixture of two or more subcategories of skin and subcutaneous tissue problems and one respiratory symptom (eczema, dermatitis, acne, psoriasis, atopic skin, dry skin, skin spots, itching, tickly, burning throat, back, legs, nose, eyes, arms, and/or coughing) when the BMI was 30 to 34.9 (class I obesity) (25%) than when the BMI was 18.5 to 24.9 (normal weight) (20.8%) (χ^2^ (4) = 41.11; *p* < 0.001; η^2^ = 0.93). Class II obesity subjects reported mostly a single category (and not a mixture) of skin and subcutaneous tissue (eczema, dermatitis, acne, psoriasis, atopic skin, dry skin) (66.7%); making it not suitable enough to merge class I and class II obesity for statistical analysis. Similarly, there was a significantly higher frequency of subjects who reported signs and symptoms of the subcategory of abdominal boating and/or abdominal pain (18%) in overweight than in subjects of normal weight (15.4%) (χ^2^ (4) = 14; *p* = 0.007; η^2^ = 0.58). Furthermore, there was a significantly higher frequency of subjects who reported signs and symptoms of the subcategory of pain and heaviness (back, legs, arms, lower back, facial, jaw, and/or joint) (37.5%) in overweight than in subjects of normal weight (28.3%) (χ^2^ (5) = 24.49; *p* < 0.001; η^2^ = 0.72). 

Regarding the different reported ‘number of signs and symptoms’ in men and women, 22% of women reported a mean of four self-reported signs and symptoms versus 14.5% of men. A total of 21% of men reported a mean of one self-reported sign or symptom. 

Additionally, signs and symptoms of four different groups of age ranges were analyzed (20–34, 35–49, 50–64, and 65–79-year-old groups) and significant differences were found between age ranges and NS problems. The 50–64-year-old group reported mostly pain and heaviness (back, legs, arms, lower back, facial, jaw, and/or joint). Meanwhile, the 20–34-year-old group mostly reported migraines, dizziness, headache, and/or stress (χ^2^ (5) = 17.40; *p* = 0.004; η^2^ = 0.17).

### 3.3. Food-Specific Immunoglobulin G_4_ Antibody Reactions

**Figure 1:**
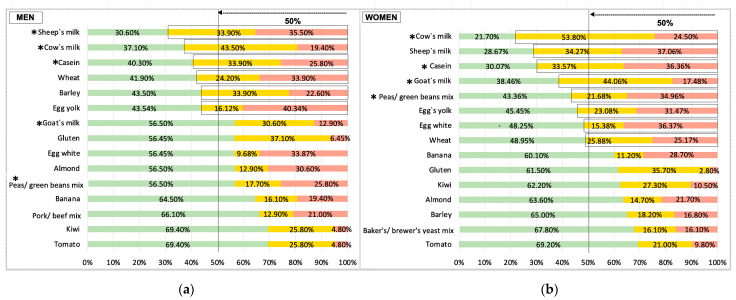
Food-specific IgG_4_ AbRs in Spanish men and women. (**a**) Prevalence of food-specific IgG_4_ AbRs in men, (**b**) prevalence of food-specific IgG_4_ AbRs in women. Green color: Level 1, low, food-specific IgG_4_ AbRs. Yellow color: Level 2, moderate, food-specific IgG_4_ AbRs. Red color: Level 3, high, food-specific IgG_4_ AbRs. * *p* < 0.05.

The most prevalent food-specific IgG_4_ AbRs are shown in Figure 1. The positive population response (≥50%, equal or greater than 50% of levels 2 + 3 IgG_4_ AbRs) in men was sheep’s milk (69.40%), cow’s milk (62.90%), casein (59.70%), wheat (58.10%), barley (56.50%), and egg yolk (56.46%). However, in women, the positive population response was cow’s milk (78.30%), sheep’s milk (71.33%), casein (69.93%), goat’s milk (61.54%), legume mix (peas/green beans mix) (56.64%), egg yolk (54.55%), egg white (51.75%), and wheat (51.05%). The positive response had more food, beverages, and allergen mix involved in women than in men, eight vs. six foodstuffs. 

When comparing sex in Level 3 of IgG_4_ AbRs, the following were significantly higher for women than men: casein (Z = 1.68; *p* = 0.047; η^2^ = 0.014); cow’s milk (Z = 1.94; *p* = 0.026; η^2^ = 0.018); goat´s milk (Z = 2.18; *p* = 0.015; η^2^ = 0.023); and legume mix (peas/green beans mix) (Z = 1.68; *p* = 0.046; η^2^ = 0.014). Similarly, when comparing sex along Level 2 of IgG_4_ AbRs, goat’s milk IgG_4_ AbRs was also significantly higher for women than men (Z = 2.18; *p* = 0.029; η^2^ = 0.023) (Figure 1). 

There were some foodstuffs that directly correlated with each other. As expected, gluten allergen correlated with the level of IgG_4_ AbRs against wheat, rye, barley (all *p* < 0.001), and oats (*p* = 0.037). The variable ‘number of signs and symptoms’ had different correlations with food-specific IgG_4_ AbRs: positive correlations were found with grain mix A (buckwheat/amaranth/quinoa mix) (*p* = 0.035), as well as, with soy (*p* = 0.007) and pineapple (*p* = 0.04), but a negative correlation was found with fruit mix A (lemon/orange mix) (*p* = 0.007). 

Multiple comparison tests (MCTs) were performed and revealed that the level of IgG_4_ AbRs against pineapple was higher (Levels 2 and 3) when subjects presented from 3 to 5 self-reported ‘signs and symptoms’ related to skin and subcutaneous tissue, digestive, or NS problems (all *p* = 0.036). For the rest of the possible comparisons, no significant differences were found (*p* > 0.05).

#### 3.3.1. Food-Specific IgG_4_ AbRs and Age

Food-specific IgG_4_ AbRs between the four studied age range groups are shown in Table 2. Seventeen most prevalent foodstuff IgG_4_ AbRs were analyzed. When evaluating Level 3 positive responses, subjects belonging to the 20–34-year-old group showed a higher prevalence of casein IgG_4_ AbRs than subjects from all the other age range groups. Furthermore, significant differences were found between subjects from the 20–34 and the 35–49-year-old group (χ^2^ (3) = 12.99; *p* = 0.005; η^2^ = 0.06). Similarly, subjects from the 35–49-year-old group had a higher prevalence of egg white IgG_4_ AbRs compared to the subjects from all the other age range groups, but significant differences were found between subjects from the 35–49 and the 50–64-year-old group (χ^2^ (3) = 8.32; *p* = 0.040; η^2^ = 0.04).

#### 3.3.2. Food-Specific IgG_4_ AbRs and Symptomatology

**Figure 2:**
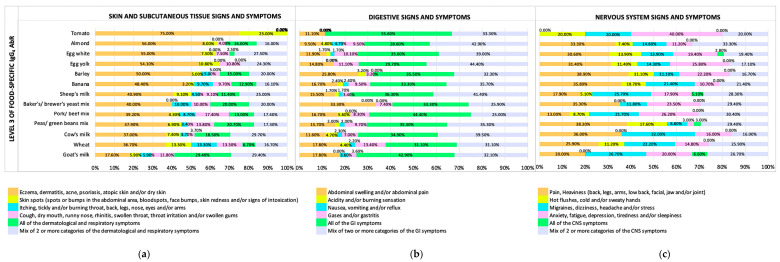
Symptomatology profile of subjects with Level 3 of food-specific IgG_4_ AbRs. (**a**) Skin and subcutaneous tissue profile of subjects with Level 3 of food-specific IgG_4_ AbRs. (**b**) Digestive profile of subjects with Level 3 of food-specific IgG_4_ AbRs. (**c**) Nervous system profile of subjects with Level 3 of food-specific IgG_4_ AbRs.

Figure 2 shows the frequency of self-reported signs and symptoms of subjects when presenting positive Level 3 of food-specific IgG_4_ AbRs against the most prevalent foodstuffs in the studied sample. 

Skin and subcutaneous tissue self-reported signs and symptoms (eczema, dermatitis, acne, psoriasis, atopic skin, and/or dry skin) were the most frequent when subjects had Level 3 of food-specific IgG_4_ AbRs against tomato (75%), almond (56%), egg white (55%), and egg yolk (54.10%). Likewise, all digestive self-reported signs and symptoms (abdominal bloating, and/or abdominal pain, acidity and/or burning sensation, nausea, vomiting and/or reflux, gas, and/or gastritis) were the most frequent when subjects had Level 3 of food-specific IgG_4_ AbRs against tomato (55.60%), egg yolk (44.40%), and meat mix A (pork/beef mix, 44.40%). Finally, NS self-reported signs and symptoms (anxiety, fatigue, depression, tiredness, and/or sleepiness) were the most frequent when subjects had Level 3 of food-specific IgG_4_ AbRs against tomato (40%), followed by pain and heaviness (back, legs, arms, lower back, facial, jaw, and/or joint) frequent signs and symptoms when subjects had Level 3 of food-specific IgG_4_ AbRs against legume mix (peas/green beans mix) (38.20%).

Positive (Level 3) food-specific IgG_4_ AbRs against barley had a symptomatology profile consisting of mostly 50% of skin and subcutaneous tissue self-reported signs and symptoms (eczema, dermatitis, acne, psoriasis, atopic skin, and/or dry skin); 35.50% of digestive signs and symptoms (abdominal bloating and/or abdominal pain, acidity, and/or burning sensation, nausea, vomiting and/or reflux, gas, and/or gastritis over the other types of GI signs and symptoms); and 38.90% of NS self-reported signs and symptoms (pain and heaviness (back, legs, arms, lower back, facial, jaw, and/or joint)).

Positive (Level 3) food-specific IgG_4_ AbRs against tomato had a symptomatology profile covering mostly 75% of skin and subcutaneous tissue (eczema, dermatitis, acne, psoriasis, atopic skin, and/or dry skin); 55.60% of digestive (abdominal bloating, and/or abdominal pain, acidity, and/or burning sensation, nausea, vomiting, and/or reflux, gas and/or gastritis); and 40% of NS self-reported signs and symptoms (anxiety, fatigue, depression, tiredness, and/or sleepiness).

The skin and subcutaneous tissue, and the NS self-reported signs and symptoms explained 25% of the variance of total IgG_4_ Ab reaction count. However, when the age range variable was adjusted as a covariate, both symptomatology variables explained 30% of the variance of the total IgG_4_ Ab reaction count. Significance improved from 0.239 to 0.149 but no statistical differences were found between symptomatology and the total IgG_4_ Ab reaction count.

## 4. Discussion

ARFS currently represent a growing global health concern. Thus, their attributable signs and symptoms, as well as possible related biomarkers, are of crucial interest for the development of research and evaluation. The study of which foods are causal of specific novel immunoglobulins is challenging since foods can be a trigger, but there are many additional triggers, including other variables, such as infection, presence of other diseases, food frequency consumption, dietary intake, crosslinking reactions with other foodstuffs, or even environmental allergens [26]. In the present study, IgG_4_ markers have been investigated as a supporting tool for the current IgE, oral challenges, and other currently used tests for the evaluation and research of ARFS. This marker has been suggested as a complementary marker when measuring IgE, as well as during FA treatment [27]. The present study analyzed a sample of 205 Spanish adults with self-reported signs and symptoms attributed to FA or FI. The most frequent self-reported signs and symptoms were related to skin and subcutaneous tissue (eczema, psoriasis, dermatitis, acne). The skin is the most frequently affected target organ in ARFS and has been reported in the past two decades to be around 40 to 60% for type I allergy (food-specific IgE AbRs) and a generalized eczematous rash or dyshidrosiform reactions of the fingers, palms, and soles for delayed-type reactions (IgG_4_ AbRs) [28]. The second most common type of self-reported signs and symptoms in this study was related to the digestive system. Previous research in participants who claimed to have a FI has reported high levels of IgG_4_ AbRs with signs and symptoms related to the GI tract, such as abdominal inconvenience, GI discomfort, and other mainly characteristic features of gut permeability and their participation in intestinal inflammatory and metabolic reactions [29]. The last prevalent type of self-reported signs and symptoms of the present study was related to the NS (depression, anxiety, fatigue, and lack of energy). According to a related published research study, [30] participants with suspicion of ARFS have also pointed out lack of energy as one of the most reported signs and symptoms.

The study of the prevalence of food-specific IgG_4_ AbRs could contribute to a clearer idea of some delayed-type ARFS and diseases related to immunoglobulin G_4_, such as IBS, ulcerative colitis (UC), or Crohn’s disease (CD). A European study investigating the presence of food-specific IgG_4_ AbRs in participants with IBS has reported a high prevalence of reactions against milk, egg, wheat, beef, pork, and lamb [31], which are among the 17 most prevalent foodstuffs in the present study. 

Another study conducted in Saudi Arabia with participants presenting allergy signs and symptoms also determined similar prevalent food-specific IgG AbRs, even though the evaluated AbRs were not specifically Subclass 4 (IgG_4_), as in this present study [12]. Among 11 prevalent food-specific IgG AbRs, 6 of them were against similar food allergens: brewer’s yeast, wheat, pea, egg white, barley, and cow’s milk when compared to the present study. Except for brewer’s yeast, prevalence in men was lower for the rest of similar prevalent food-specific IgG AbRs than those found in the present study. However, in women, food-specific IgG AbRs were higher against brewer’s yeast, wheat, pea, egg white, and barley, and lower against cow’s milk. The fact that the prevalence between women is slightly close, except for brewer’s yeast, considering different types of samples and diet but similar symptomatology related to ARFS, supports the promising clinical reproducibility of IgG_4_-mediated ARFS symptomatology [12,29,31,32,33] and reinforces the link between a symptomatology profile with likely specific foodstuffs [5,34,35].

Regarding the variable of age, a Saudi Arabian study was analyzed using 2 groups: 20–39-year-olds and 40–80-year-olds [12], showing that food-specific IgG AbRs against all allergens decreased in prevalence according to age. Compared to the present study, age was analyzed using a wider distribution of ages: 20–34, 35–49, 50–64, and 65–79-year-old groups observing that IgG_4_ AbRs against dairy-related allergens such as goat’s milk might change depending on the age range, showing a similar prevalence in all age ranges except for the 35–49-year-old group, which had the lowest prevalence. Similarly, for IgG_4_ AbRs against egg white, the prevalence was similar in all age ranges, except for the 35–49-year-old group, which had the highest prevalence. In the present study, the age range that marked the most difference was the 35–49-year-old group and not the youngest age range as in the Saudi Arabian study. Age distribution and the specific characteristics of the test (sensitivity, specificity, antigen concentration, and levels of detection) might be one of the main reasons for this contrast. Using the AESKUCARE-T2FA^®^ POC test kit in the present study, steps such as incubation, washing, and staining were performed using a ready-to-use main reaction plate and reagents, decreasing the risk of practitioner/technician error. Usually, young adults (35–49 y.o.) tend to be the most common age range affected in Europe regarding FA and ARFS-related symptoms [36]. In the older group (65–79-year-old group), there were no cases of moderate Level 2 of IgG_4_ AbRs against legume mix (peas/green beans mix) and banana. 

Comparably, a South Korean study that evaluated food-specific IgE/IgG_4_ AbRs against milk, egg white, wheat, and soybean, explained the importance of allergen-specific IgE/IgG_4_ as a tool to investigate the mechanism of FA in atopic dermatitis [32]. The underlying mechanism of ARFS involves a combination of multiple paths, including immediate reactions (type I, IgE mediated) and delayed-type reactions (IgE, IgM, IgA, IgG antibodies and subclasses) [37]. 

The present study had a defined sample with the body composition characteristics of an individual with self-reported signs and symptoms attributed to ARFS. In other words, subjects with diet and lifestyle struggles usually lead to sedentarism, obesity, inflammation, and even difficulty digesting some of the most common food products and components of the MD diet (e.g., milk, legume, tomato, gluten). Gluten and IgG reactions might be associated with systemic inflammation [34] and contribute to the pathophysiology of IBS [38]. Furthermore, the literature suggests that overweight and obese subjects tend to have a higher frequency of GI comorbidity [39]. Barley, and its components, also appear to play an important role in obesity [40,41].

According to an article on precision medicine in FA [42], no participants with the same FA are expected to show the same reported signs and symptoms. In the case of a similar positive FA test (e.g., food-specific IgE AbRs), the natural history may be different. In another study, wheat sensitivity is mainly attributed only to GI reported signs and symptoms [43]. However, in the present study, it was also related to a dermatological and NS profile. 

The close relationship between PA and the immune system is exposed in the present study when analyzing whether participants meet the WHO recommendations for PA [44] and correlating it with the most prevalent food-specific IgG_4_ AbRs. Negative associations were observed between the achievement of the minimum WHO requirements of PA and IgG_4_ AbRs against barley and between low PA and IgG_4_ AbRs against gluten and sheep’s milk. The fact that PA might increase gut and colon permeability and inflammation could explain the amount of positive correlations between PA and food-specific IgG_4_ AbRs [45].

Legume mix (peas/green beans mix) was the only food allergen missing from the EU list of mandatory declaration of all prevalent allergens in the present study. Peas have been closely related to FA in the Spanish population for several decades [46] and green beans have been associated with frequent signs and symptoms of FA and FI such as urticaria, asthma, or rhinitis [47,48]. European regulatory agencies usually base decisions on the annual reported cases of ARFS, which are mostly type I reactions (anaphylactic or other immediate reactions) [49], leaving out delayed-type reactions that might also affect the Spanish population in terms of skin and subcutaneous tissue, digestive and NS problems.

### 4.1. Main Findings

There are self-reported signs and symptoms of the skin and subcutaneous tissue, digestive, and NS in a Spanish sample of adults aged 20 to 79 years who attribute their symptomatology to ARFS (FA and FI) and live in the Region of Madrid. Low PA and overweight are common parameters among the studied sample. The most common positive food-specific IgG_4_ AbRs among men were mainly related to milk (cow’s milk, sheep’s milk, and casein), followed by wheat, barley, and egg yolk. And the most common among women were also mainly related to milk (cow’s milk, sheep’s milk, goat’s milk, and casein); followed by wheat, egg white, egg yolk, and legume mix (peas/green beans mix).

### 4.2. Strengths and Limitations

This study has been performed using a AESKUCARE-T2FA^®^ in vitro POC and ready-to-use test kit that decreases the technician and practitioners’ error during immunological detection practices. Data collection and clinical analysis were performed before the COVID-19 pandemic. This condition benefited the uniformity of the sample. The self-reported signs and symptoms might be less biased avoiding possible post-COVID sequelae that are still being studied. Current evidence reports that individuals who have recovered from COVID-19 may still have persistent signs and symptoms, radiological abnormalities, and compromised respiratory function [50]. Food-specific IgG_4_ AbRs have been studied to expand the scientific knowledge of its role in ARFS since there is a current unclear studied path.

As a limitation, data collection started as a regular clinical appointment without research purposes; however, the results were adjusted according to validated scales when the answers allowed it. Furthermore, there is a lack of similar published data using the same food-specific IgG_4_ AbRs panel together with symptomatology profiles for a more accurate comparison with the results of the present study. 

### 4.3. Future Research

A strong protocol containing more variables of interest is needed to maximize statistical power based on these preliminary observations. The following validated tools will be used for future ARFS research to assess the subject’s diseases, signs, symptoms, and food and beverage intake: Pathologies and Symptomatology Questionnaire associated with Adverse Reactions to Foodstuffs (PSIMP-ARFSQ-10); and Food and Beverages Frequency Consumption Questionnaire to Identify Adverse Reactions to Foodstuffs (FBFC-ARFSQ-18) [51]. Obtained results suggest interesting trends of food-specific IgG_4_ AbRs toward body composition, PA, sex, age, and symptomatology profiles that lead to continuing research in the field. Additional health parameters and variables will be considered for future similar research, such as clinical analysis (e.g., hematobiochemical) and food intake evaluations (e.g., food frequency consumption and dietary intake). Follow-up studies using validated tools will be performed to establish a consistent clinical picture of ARFS and more data investigating the role of IgG_4_ AbRs in ARFS. Food-specific IgE AbRs evaluations will be also considered for future research due to the tight relationship that they currently have with food allergies. 

## 5. Conclusions

The most frequent self-reported signs and symptoms in the studied Spanish sample of adults with suspicion of ARFS included skin and subcutaneous tissue, digestive, and NS problems. Spanish adults aged 35 to 49 years may be of potential interest regarding the frequency of the symptomatology attributed to ARFS. The higher prevalence of IgG_4_ AbRs found in the studied sample was related to milk (cow’s milk, goat’s milk, sheep’s milk, and casein), followed by cereal (wheat and barley), egg (egg white and egg yolk), and legume (peas/green beans mix). Food-specific IgG_4_ AbRs are similar among men and women in terms of the type of food and beverage allergen or allergen mix (milk, egg, cereal, and legume mix), but vary in terms of its prevalence, except for legume mix (peas/green beans mix), which is only prevalent in women. Women had positive responses to a greater number of food and beverage allergens/allergen mix than men (eight versus six). Moreover, positive Level 3 of IgG_4_ AbRs against tomato had a symptomatology profile consisting of 3/4 of dermatological, more than half of GI, and 2/5 of self-reported signs and symptoms of the NS.

## Figures and Tables

**Table 1:** Sample characteristics.

	M ± SD or %			
	Total (*n* = 205)	Men (*n* = 62)	Women (*n* = 143)	Min–Max
Age	45.46 ± 14.91	47.45 ± 13.27	44.59 ± 15.53	20–79
Body composition				
Height (m)	1.67 ± 0.09	1.76 ± 0.06	1.63 ± 0.07	1.50–1.92
Weight (kg)	73.39 ± 16.12	82.92 ± 15.63	69.26 ± 14.54	42.30–130.60
Fat-free mass (kg)	47.72 ± 10.12	52.93 ± 10.65	45.46 ± 9.03	30.71–74.75
Muscular mass (%)	59.28 ± 12.20	56.96 ± 13.29	60.28 ± 11.60	26.91–97.12
Bone mass (kg)	2.77 ± 0.76	3.08 ± 1.23	2.63 ± 0.34	1.90–12.11
Fat mass (%)	33.17 ± 9.07	32.46 ± 9.43	33.47 ± 8.92	10.80–60.30
Body mass index	26.32 ± 4.99	28.05 ± 4.93	25.56 ± 4.84	16.90–43.30
Body mass index				
<18.5 underweight (%)	1.95	0	2.80	--
18.5–24.9 normal weight (%)	41.46	29.03	46.85	--
25.0–29.9 overweight (%)	31.71	30.65	32.17	--
30.0–34.9 class I obesity (%)	20.00	30.65	15.38	--
35.0–39.9 class II obesity (%)	4.39	9.68	2.10	--
>40 class III obesity (%)	0.49	0	0.70	--
Physical activity (IPAQ-SF ^1^)				
Low (%)	87.30	95.20	83.90	--
Moderate (%)	12.70	4.80	16.10	--
High (%)	0	0	0	--
Sitting time (min/week)	2577.37 ± 943.82	2499.68 ± 978.15	2611.05 ± 930.03	1260–5040
Sitting time (h/week)	42.96 ± 15.73	41.66 ± 16.30	43.52 ± 15.50	21–84
Symptomatology				
Dermatological (%)	43.27	37.40	45.50	--
Digestive or GI ^2^ (%)	40.74	47.90	38.60	--
NS ^3^ (%)	33.33	52.40	34.50	--

^1^ IPAQ-SF: International Physical Activity Questionnaire Short Form; ^2^ GI: gastrointestinal; ^3^ NS: nervous system.

**Table 2:** Food-specific IgG_4_ AbRs levels in Spanish adults of different age range groups.

Distribution of Food-Specific IgG_4_ AbRs in Men and Women in Different Age Range Groups										
**Age range (20–34)**										
		**Total**				**Men**			**Women**	
		***n* = 57**				***n* = 13**			***n* = 44**	
Level of IgG_4_ AbRs	**1**	**2**	**3**	*p* Values	**1**	**2**	**3**	**1**	**2**	**3**
Casein	24.60%	24.60%	50.80%	NS	15.40%	30.80%	53.80%	27.30%	22.70%	50.00%
Sheep’s milk	19.30%	33.30%	47.40%	NS	0.00%	46.20%	53.80%	25.00%	29.50%	45.50%
Egg white	52.60%	15.80%	31.60%	NS	53.80%	23.10%	23.10%	52.30%	13.60%	34.10%
Peas/green beans mix	45.60%	22.80%	31.60%	NS	53.80%	23.10%	23.10%	43.20%	22.70%	34.10%
Wheat	45.60%	22.80%	31.60%	NS	23.00%	38.50%	38.50%	52.30%	18.20%	29.50%
Banana	59.60%	12.30%	28.10%	NS	69.20%	7.70%	23.10%	56.90%	13.60%	29.50%
Cow’s milk	15.80%	56.10%	28.10%	NS	7.70%	61.50%	30.80%	18.20%	54.50%	27.30%
Egg yolk	43.90%	29.80%	26.30%	NS	30.70%	38.50%	30.80%	47.70%	27.30%	25.00%
Pork/beef mix	61.40%	14.00%	24.60%	NS	61.50%	0.00%	38.50%	61.40%	18.20%	20.50%
Lamb or mutton	68.40%	8.80%	22.80%	NS	61.50%	7.70%	30.80%	70.50%	9.10%	20.50%
Almond	64.90%	15.80%	19.30%	NS	76.90%	7.70%	15.40%	61.40%	18.10%	20.50%
Barley	57.90% a	24.60% b	17.50% ab	0.06	23.00%	46.20%	30.80%	68.20%	18.20%	13.60%
Goat’s milk	36.80%	49.20%	14.00%	NS	38.50%	53.80%	7.70%	36.40%	47.70%	15.90%
Baker’s/brewer’s yeast mix	73.70%	15.80%	10.50%	NS	84.60%	0.00%	15.40%	70.50%	20.50%	9.00%
Tomato	73.70%	19.30%	7.00%	NS	53.80%	38.50%	7.70%	79.60%	13.60%	6.80%
Kiwi	77.20%	17.50%	5.30%	NS	84.60%	15.40%	0.00%	75.00%	18.20%	6.80%
Gluten	57.90%	40.40%	1.70%	NS	38.50%	61.50%	0.00%	63.60%	34.10%	2.30%
**Age range** **(35–49)**										
		**Total**				**Men**			**Women**	
		***n* = 67**				***n* = 22**			***n* = 45**	
Level of IgG_4_ AbRs	**1**	**2**	**3**	*p* Values	**1**	**2**	**3**	**1**	**2**	**3**
Casein	35.80%	43.30%	20.90%	NS	45.50%	40.90%	13.60%	31.20%	44.40%	24.40%
Sheep’s milk	44.80%	26.90%	28.30%	NS	50.00%	31.80%	18.20%	42.20%	24.50%	33.30%
Egg white	38.80%	11.90%	49.30%	NS	40.90%	9.10%	50.00%	37.80%	13.30%	48.90%
Peas/green beans mix	53.70%	19.40%	26.90%	NS	68.20%	18.20%	13.60%	46.70%	20.00%	33.30%
Wheat	43.30%	31.30%	25.40%	NS	36.40%	22.70%	40.90%	46.70%	35.50%	17.80%
Banana	56.70%	16.40%	26.90%	NS	72.70%	9.10%	18.20%	48.90%	20.00%	31.10%
Cow’s milk	29.90%	47.80%	22.30%	NS	40.90%	50.00%	9.10%	24.40%	46.70%	28.90%
Egg yolk	34.30%	17.90%	47.80%	NS	31.80%	4.60%	63.60%	35.60%	24.40%	40.00%
Pork/beef mix	76.10%	7.50%	16.40%	NS	77.30%	9.10%	13.60%	75.60%	6.70%	17.70%
Lamb or mutton	80.60%	6.00%	13.40%	NS	86.40%	4.50%	9.10%	77.80%	6.70%	15.50%
Almond	65.70%	11.90%	22.40%	NS	72.70%	4.50%	22.70%	62.20%	15.60%	22.20%
Barley	49.20%	23.90%	26.90%	NS	45.50%	27.30%	27.20%	51.10%	22.20%	26.70%
Goat’s milk	55.20%	26.90%	17.90%	NS	68.20%	22.70%	9.10%	48.90%	28.90%	22.20%
Baker’s/brewer’s yeast mix	71.60% a	11.90% ab	16.50% b	0.01	90.90%	9.10%	0.00%	62.30%	13.30%	24.40%
Tomato	65.70%	23.90%	10.40%	NS	72.70%	18.20%	9.10%	62.20%	26.70%	11.10%
Kiwi	64.10% a	29.90% b	6.00% ab	0.001	90.90%	9.10%	0.00%	51.10%	40.00%	8.90%
Gluten	61.20%	34.30%	4.50%	NS	45.50%	45.50%	9.00%	68.90%	28.90%	2.20%
**Age range (50–64)**										
		**Total**				**Men**			**Women**	
		***n* = 59**				***n* = 20**			***n* = 39**	
Level of IgG_4_ AbRs	**1**	**2**	**3**	*p* Values	**1**	**2**	**3**	**1**	**2**	**3**
Casein	35.60%	32.20%	32.20%	NS	45.00%	30.00%	25.00%	30.80%	33.30%	35.90%
Sheep’s milk	25.40%	39.00%	35.60%	NS	40.00%	25.00%	35.00%	17.90%	46.20%	35.90%
Egg white	61.00%	13.60%	25.40%	NS	70.00%	5.00%	25.00%	56.50%	17.90%	25.60%
Peas/green beans mix	44.10%	27.10%	28.80%	NS	50.00%	20.00%	30.00%	41.00%	30.80%	28.20%
Wheat	52.60%	23.70%	23.70%	NS	55.00%	25.00%	20.00%	51.30%	23.10%	25.60%
Banana	67.80%	13.60%	18.60%	NS	60.00%	35.00%	5.00%	71.80%	2.60%	25.60%
Cow’s milk	28.80%	50.80%	20.40%	NS	45.00%	35.00%	20.00%	20.50%	59.00%	20.50%
Egg yolk	54.30%	16.90%	28.80%	NS	55.00%	15.00%	30.00%	53.80%	17.90%	28.30%
Pork/beef mix	54.30%	23.70%	22.00%	NS	65.00%	20.00%	15.00%	48.80%	25.60%	25.60%
Lamb or mutton	61.00%	20.40%	18.60%	NS	70.00%	20.00%	10.00%	56.40%	20.50%	23.10%
Almond	57.70%	16.90%	25.40%	NS	40.00%	30.00%	30.00%	66.70%	10.30%	23.00%
Barley	61.00%	27.10%	11.90%	NS	50.00%	40.00%	10.00%	66.70%	20.50%	12.80%
Goat’s milk	39.00%	45.80%	15.20%	NS	55.00%	30.00%	15.00%	30.80%	53.80%	15.40%
Baker’s/brewer’s yeast mix	72.90%	11.90%	15.20%	NS	65.00%	10.00%	25.00%	76.90%	12.80%	10.30%
Tomato	71.20%	20.30%	8.50%	NS	75.00%	25.00%	0.00%	69.30%	17.90%	12.80%
Kiwi	55.90%	28.80%	15.30%	NS	84.60%	15.40%	0.00%	64.20%	17.90%	17.90%
Gluten	57.60%	35.60%	6.80%	NS	75.00%	15.00%	10.00%	48.70%	46.20%	5.10%
**Age range (65–79)**										
		**Total**				**Men**			**Women**	
		***n* = 22**				***n* = 7**			***n* = 15**	
Level of IgG_4_ AbRs	**1**	**2**	**3**	*p* Values	**1**	**2**	**3**	**1**	**2**	**3**
Casein	40.90%	31.80%	27.30%	NS	57.10%	28.60%	14.30%	33.30%	33.40%	33.30%
Sheep’s milk	18.20%	45.50%	36.30%	NS	0.00%	42.90%	57.10%	26.70%	46.60%	26.70%
Egg white	54.50%	13.70%	31.80%	NS	71.40%	0.00%	28.60%	46.70%	20.00%	33.30%
Peas/green beans mix	40.90%	0.00%	59.10%	NS	42.90%	0.00%	57.10%	40.00%	0.00%	60.00%
Wheat	45.50%	18.20%	36.30%	NS	57.10%	0.00%	42.90%	40.00%	26.70%	33.30%
Banana	63.60%	0.00%	36.40%	NS	42.90%	0.00%	57.10%	73.30%	0.00%	26.70%
Cow’s milk	36.30%	45.50%	18.20%	NS	57.10%	14.30%	28.60%	26.70%	60.00%	13.30%
Egg yolk	54.50%	18.20%	27.30%	NS	71.40%	14.30%	14.30%	46.70%	20.00%	33.30%
Pork/beef mix	50.00%	22.70%	27.30%	NS	42.80%	28.60%	28.60%	53.30%	20.00%	26.70%
Lamb or mutton	59.10%	22.70%	18.20%	NS	42.80%	28.60%	28.60%	66.70%	20.00%	13.30%
Almond	50.00% a	9.10% ab	40.90% b	0.009	14.30%	0.00%	85.70%	66.70%	13.30%	20.00%
Barley	81.80%	4.50%	13.70%	NS	57.10%	14.30%	28.60%	93.30%	0.00%	6.70%
Goat’s milk	40.90%	40.90%	18.20%	NS	57.10%	14.30%	28.60%	33.30%	53.40%	13.30%
Baker’s/brewer’s yeast mix	68.20%	13.60%	18.20%	0.036	100.00%	0.00%	0.00%	53.30%	20.00%	26.70%
Tomato	63.70%	31.80%	4.50%	NS	71.40%	28.60%	0.00%	60.00%	33.30%	6.70%
Kiwi	54.50%	36.40%	9.10%	NS	57.10%	28.60%	14.30%	53.30%	40.00%	6.70%
Gluten	68.20%	31.80%	0.00%	NS	71.40%	28.60%	0.00%	66.70%	33.30%	0.00%

Values bearing different letters were significantly different (a ≠ b). NS: not statistically significant.

## Data Availability

The supporting reported dataset of this study can be found in The Multidisciplinary Data Repository of the Madroño Consortium, ‘e-cienciaDatos’ at (https://doi.org/10.21950/NA8QKC), accessed on 16 October 2023, reference: (10.21950/NA8QKC) [52].
